# Supplementary material for: HIVprotI: an integrated web based platform for prediction and design of HIV proteins inhibitors
Source: J Cheminform. 2018 Mar 9;10:12. doi: 10.1186/s13321-018-0266-y (PMC5845081; doi:10.1186/s13321-018-0266-y)
Supplement: Supplementary file 2 — Additional file 2. Source code of HIVProtI web server. [file 13321_2018_266_MOESM2_ESM.zip › HIVprotI_Source-code/hivprotI/weka-3-6-6/doc/weka/gui/HierarchyPropertyParser.html]

HierarchyPropertyParser


---


|  |  |  |
| --- | --- | --- |
| **Overview** \| **Package** \| **Class** \| **Tree** \| **Deprecated** \| **Index** \| **Help** \| **Weka's home** | |  |
| **PREV CLASS**   **NEXT CLASS** | **FRAMES**    **NO FRAMES**     **All Classes** |  |
| SUMMARY: NESTED \| FIELD \| CONSTR \| METHOD | DETAIL: FIELD \| CONSTR \| METHOD |  |


---


## weka.gui Class HierarchyPropertyParser

```
java.lang.Object
  weka.gui.HierarchyPropertyParser
```

**All Implemented Interfaces:**: java.io.Serializable

---

``` public class HierarchyPropertyParser extends java.lang.Object implements java.io.Serializable ```

This class implements a parser to read properties that have
a hierarchy(i.e. tree) structure. Conceptually it's similar to
the XML DOM/SAX parser but of course is much simpler and
uses dot as the seperator of levels instead of back-slash.  
It provides interfaces to both build a parser tree and traverse
the tree.   
Note that this implementation does not lock the tree when different
threads are traversing it simultaneously, i.e. it's NOT synchronized
and multi-thread safe. It is recommended that later implementation
extending this class provide a locking scheme and override the
functions with the "synchronized" modifier (most of them are
goToXXX() and information accessing functions).

**Version:**
:   $Revision: 7059 $

**Author:**
:   Xin Xu (xx5@cs.waikato.ac.nz)

**See Also:**: Serialized Form

---

**Constructor Summary**

`HierarchyPropertyParser()`
  
          Default constructor

`HierarchyPropertyParser(java.lang.String p,
java.lang.String delim)`
  
          Constructor that builds a tree from the given property with
the given delimitor


| **Method Summary** | |
| --- | --- |
| `void` | `add(java.lang.String property)`             Add the given item of property to the tree |
| `void` | `build(java.lang.String p, java.lang.String delim)`             Build a tree from the given property with the given delimitor |
| `java.lang.String[]` | `childrenValues()`             The value in the children nodes. |
| `boolean` | `contains(java.lang.String string)`             Whether the HierarchyPropertyParser contains the given string |
| `java.lang.String` | `context()`             The context of the current node, i.e. |
| `int` | `depth()`             Get the depth of the tree, i.e. |
| `java.lang.String` | `fullValue()`             The full value of the current node, i.e. |
| `int` | `getLevel()`             Get the level of current node. |
| `java.lang.String` | `getSeperator()`             Get the seperator between levels. |
| `java.lang.String` | `getValue()`             Get the value of current node |
| `boolean` | `goDown(java.lang.String path)`             Go to a certain node of the tree down from the current node according to the specified relative path. |
| `boolean` | `goTo(java.lang.String path)`             Go to a certain node of the tree according to the specified path Note that the path must be absolute path from the root. |
| `void` | `goToChild(int pos)`             Go to one child node from the current position in the tree according to the given position |
| `boolean` | `goToChild(java.lang.String value)`             Go to one child node from the current position in the tree according to the given value   If the child node with the given value cannot be found it returns false, true otherwise. |
| `void` | `goToParent()`             Go to the parent from the current position in the tree If the current position is the root, it stays there and does not move |
| `void` | `goToRoot()`             Go to the root of the tree |
| `boolean` | `isHierachic(java.lang.String string)`             Whether the given string has a hierachy structure with the seperators |
| `boolean` | `isLeafReached()`             Whether the current position is a leaf |
| `boolean` | `isRootReached()`             Whether the current position is the root |
| `static void` | `main(java.lang.String[] args)`             Tests out the parser. |
| `int` | `numChildren()`             The number of the children nodes. |
| `java.lang.String` | `parentValue()`             The value in the parent node. |
| `int` | `search(java.util.Vector vct, java.lang.String target)`             Helper function to search for the given target string in a given vector in which the elements' value may hopefully is equal to the target. |
| `void` | `setSeperator(java.lang.String s)`             Set the seperator between levels. |
| `java.lang.String` | `showTree()`             Show the whole tree in text format |
| `java.lang.String[]` | `tokenize(java.lang.String rawString)`             Tokenize the given string based on the seperator and put the tokens into an array of strings |

**Methods inherited from class java.lang.Object**

`equals, getClass, hashCode, notify, notifyAll, toString, wait, wait, wait`

**Constructor Detail**

### HierarchyPropertyParser

```
public HierarchyPropertyParser()
```

:   Default constructor

---


### HierarchyPropertyParser

```
public HierarchyPropertyParser(java.lang.String p,
                               java.lang.String delim)
                        throws java.lang.Exception
```

:   Constructor that builds a tree from the given property with
    the given delimitor

    **Parameters:**: `p` - the given property string: `delim` - the given dilimitor **Throws:**: `java.lang.Exception`


**Method Detail**

### setSeperator

```
public void setSeperator(java.lang.String s)
```

:   Set the seperator between levels. Default is dot.

    :   **Parameters:**: `s` - the seperator symbol

---


### getSeperator

```
public java.lang.String getSeperator()
```

:   Get the seperator between levels. Default is dot.

    :   **Returns:**: the seperator symbol

---


### build

```
public void build(java.lang.String p,
                  java.lang.String delim)
           throws java.lang.Exception
```

:   Build a tree from the given property with the given delimitor

    :   **Parameters:**: `p` - the given property: `delim` - the given delimitor **Throws:**: `java.lang.Exception`

---


### add

```
public void add(java.lang.String property)
```

:   Add the given item of property to the tree

    :   **Parameters:**: `property` - the given item

---


### tokenize

```
public java.lang.String[] tokenize(java.lang.String rawString)
```

:   Tokenize the given string based on the seperator and
    put the tokens into an array of strings

    :   **Parameters:**: `rawString` - the given string **Returns:**: an array of strings

---


### contains

```
public boolean contains(java.lang.String string)
```

:   Whether the HierarchyPropertyParser contains the given
    string

    :   **Parameters:**: `string` - the given string **Returns:**: whether contains

---


### isHierachic

```
public boolean isHierachic(java.lang.String string)
```

:   Whether the given string has a hierachy structure with
    the seperators

    :   **Parameters:**: `string` - the given string

---


### search

```
public int search(java.util.Vector vct,
                  java.lang.String target)
```

:   Helper function to search for the given target string in a
    given vector in which the elements' value may hopefully is equal
    to the target. If such elements are found the first index
    is returned, otherwise -1

    :   **Parameters:**: `vct` - the given vector: `target` - the given target string **Returns:**: the index of the found element, -1 if not found

---


### goTo

```
public boolean goTo(java.lang.String path)
```

:   Go to a certain node of the tree according to the specified path
    Note that the path must be absolute path from the root.   
    For relative path, see goDown(String path).

    :   **Parameters:**: `path` - the given absolute path **Returns:**: whether the path exists, if false the current position does not move

---


### goDown

```
public boolean goDown(java.lang.String path)
```

:   Go to a certain node of the tree down from the current node
    according to the specified relative path. The path does not
    contain the value of current node

    :   **Parameters:**: `path` - the given relative path **Returns:**: whether the path exists, if false the current position does not move

---


### goToRoot

```
public void goToRoot()
```

:   Go to the root of the tree

---


### goToParent

```
public void goToParent()
```

:   Go to the parent from the current position in the tree
    If the current position is the root, it stays there and does
    not move

---


### goToChild

```
public boolean goToChild(java.lang.String value)
```

:   Go to one child node from the current position in the tree
    according to the given value   
    If the child node with the given value cannot be found it
    returns false, true otherwise. If false, the current position
    does not change

    :   **Parameters:**: `value` - the value of the given child **Returns:**: whether the child can be found

---


### goToChild

```
public void goToChild(int pos)
               throws java.lang.Exception
```

:   Go to one child node from the current position in the tree
    according to the given position   

    :   **Parameters:**: `pos` - the position of the given child **Throws:**: `java.lang.Exception` - if the position is out of range or leaf is reached

---


### numChildren

```
public int numChildren()
```

:   The number of the children nodes. If current node is leaf, it
    returns 0.

    :   **Returns:**: the number of the children nodes of the current position

---


### childrenValues

```
public java.lang.String[] childrenValues()
```

:   The value in the children nodes. If current node is leaf, it
    returns null.

    :   **Returns:**: the value in the children nodes

---


### parentValue

```
public java.lang.String parentValue()
```

:   The value in the parent node. If current node is root, it
    returns null.

    :   **Returns:**: the value in the parent node

---


### isLeafReached

```
public boolean isLeafReached()
```

:   Whether the current position is a leaf

    :   **Returns:**: whether the current position is a leaf

---


### isRootReached

```
public boolean isRootReached()
```

:   Whether the current position is the root

    :   **Returns:**: whether the current position is the root

---


### getValue

```
public java.lang.String getValue()
```

:   Get the value of current node

    :   **Returns:**: value level

---


### getLevel

```
public int getLevel()
```

:   Get the level of current node. Note the level starts from 0

    :   **Returns:**: the level

---


### depth

```
public int depth()
```

:   Get the depth of the tree, i.e. (the largest level)+1

    :   **Returns:**: the depth of the tree

---


### context

```
public java.lang.String context()
```

:   The context of the current node, i.e. the path from the
    root to the parent node of the current node, seperated by
    the seperator. If root, it returns null

    :   **Returns:**: the context path

---


### fullValue

```
public java.lang.String fullValue()
```

:   The full value of the current node, i.e. its context + seperator
    + its value. For root, only its value.

    :   **Returns:**: the context path

---


### showTree

```
public java.lang.String showTree()
```

:   Show the whole tree in text format

    :   **Returns:**: the whole tree in text format

---


### main

```
public static void main(java.lang.String[] args)
```

:   Tests out the parser.

    :   **Parameters:**: `args` - should contain nothing


---


|  |  |  |
| --- | --- | --- |
| **Overview** \| **Package** \| **Class** \| **Tree** \| **Deprecated** \| **Index** \| **Help** \| **Weka's home** | |  |
| **PREV CLASS**   **NEXT CLASS** | **FRAMES**    **NO FRAMES**     **All Classes** |  |
| SUMMARY: NESTED \| FIELD \| CONSTR \| METHOD | DETAIL: FIELD \| CONSTR \| METHOD |  |


---
